# Formation of Ge-Sn nanodots on Si(100) surfaces by molecular beam epitaxy

**DOI:** 10.1186/1556-276X-6-85

**Published:** 2011-01-12

**Authors:** Vladimir Mashanov, Vladimir Ulyanov, Vyacheslav Timofeev, Aleksandr Nikiforov, Oleg Pchelyakov, Ing-Song Yu, Henry Cheng

**Affiliations:** 1 A.V. Rzhanov Institute of Semiconductor Physics SB RAS, Lavrentyev Avenue, 13, Novosibirsk 630090, Russia; 2 Center for Condensed Matter Sciences and Graduate Institute of Electronic Engineering, National Taiwan University, Taipei, 106, Taiwan, R.O.C

## Abstract

The surface morphology of Ge_0.96_Sn_0.04_/Si(100) heterostructures grown at temperatures from 250 to 450°C by atomic force microscopy (AFM) and scanning tunnel microscopy (STM) *ex situ *has been studied. The statistical data for the density of Ge_0.96_Sn_0.04 _nanodots (ND) depending on their lateral size have been obtained. Maximum density of ND (6 × 10^11 ^cm^-2^) with the average lateral size of 7 nm can be obtained at 250°C. Relying on the reflection of high energy electron diffraction, AFM, and STM, it is concluded that molecular beam growth of Ge_1-*x*_Sn*_x _*heterostructures with the small concentrations of Sn in the range of substrate temperatures from 250 to 450°C follows the Stranski-Krastanow mechanism. Based on the technique of recording diffractometry of high energy electrons during the process of epitaxy, the wetting layer thickness of Ge_0.96_Sn_0.04 _films is found to depend on the temperature of the substrate.

## Introduction

Self-assembled Ge-Sn nanodots (ND) are considered to be a possible candidate for direct band gap materials and have high potential for a variety of applications due to their compatibility with Si technology [1,2]. Ge-Sn ND have been grown on Si substrates by methods of molecular beam epitaxy (MBE) covered with ultrathin SiO_2 _films [3,4]. A quantum-confinement effect in individual Ge_1-*x*_Sn*_x _*ND on Si(111) surfaces covered with ultrathin SiO_2 _films was observed using scanning tunneling spectroscopy at room temperature [5]. Strong 1.5 μm photoluminescence from Si-capped Ge_1-*x*_Sn*_x _*ND on Si(100) surfaces has also been observed by Nakamura et al. [3].

The epitaxial growth of Ge_1-*x*_Sn*_x _*alloys is complicated because of a big lattice mismatch (15%) between Sn and Ge, small equilibrium solid solubility of Sn in Ge (< 0.5 at.%), and a tendency for Sn surface segregation [6-8]. MBE as a non-equilibrium growth technique can overcome the former two difficulties, but the surface segregation of Sn still occurs at typical growth temperatures more than 300°C [6,9], especially for higher Sn concentration growth.

Until now, the initial stages of the epitaxial process of Ge-Sn layers on clean Si(100) surfaces from molecular beams have been scarcely reported in the literature. In particular, the growth mechanism has not been investigated. However, the growth processes in heterosystem Ge_1-*x*_Si*_x_*/Si(100) have been studied sufficiently. The epitaxy of germanium on silicon surfaces (100) turned out to follow the Stranski-Krastanow (SK) mechanism [10]. The SK model supposes that a uniformly strained film (the wetting layer) grows pseudomorphically on the substrate below some thickness of Ge or Ge_1-*x*_Si*_x_*. As its thickness increases, the islands appear on the wetting layer. Hut-clusters with faceted planes of the type {510} followed by dome-clusters with faceted {311} and {201} planes originate [11].

The technique of reflection of high energy electron diffraction (RHEED) has been used to monitor the evolution of the surface structure during the growth of the solid solution Ge_0.96_Sn_0.04 _on Si(100). RHEED is the most informative method of investigating *in situ *MBE heterostructures. As well as the previous researches [12], the authors analyzed the intensity of RHEED patterns in the growth of Ge-Sn layers. The analysis allows us to measure the wetting layer thickness [i.e., the thickness at which transition from two- (2D) to three-dimensional (3D) growth takes place] depending on the growth temperature.

The purpose of this article is to study the initial growing stages of Ge-Sn alloys on Si(100) surfaces and the distribution of Ge-Sn ND at the temperature range from 150 to 450°C by the technique of RHEED *in situ*, atomic force microscopy (AFM), and scanning tunnel microscopy (STM) *ex situ*.

## Experimental details

Samples were grown by using a solid-source MBE machine with two pyrolitic boron nitride Knudsen source cells for evaporation of germanium and tin, as well as by an electron beam evaporator for silicon. Analytic equipment in the growth chamber included a quartz thickness monitor and a high energy electron (20 kV) diffractometer. Diffraction patterns were performed during the growth by using CCD camera which permitted us to have both RHEED images on the whole and the fragments of the diffraction patterns at the rate of 10 frames per second. Ge growth rate was 0.09 nm/s, and Sn growth rate was equal to 3.8 × 10^-4 ^nm/s, which gave us the molecular beams in proportion equal to 4 at.% of Sn in Ge-Sn solid solution. Here, 4 at.% of Sn were chosen because of the large lattice mismatch among α-Sn (*a *= 0.6489 nm), Ge (*a *= 0.5658 nm), and Si (*a *= 0.5431 nm). The lattice parameter mismatch between Ge_0.96_Sn_0.04 _and Si is 4.8% theoretically, which is close in magnitude to a similar parameter of the well-studied heterostructure Ge/Si(100). The temperature of the substrates was changed from 150 to 450°C. Silicon (100) substrates were less than 0.5° disoriented. Before the Ge-Sn film started growing, the Si substrate was annealed at 1000°C, and the buffer Si layer was grown at 700°C. The micromorphology of the grown surfaces was studied by methods of AFM and STM *ex situ*.

## Results and discussion

**Figure 1:**
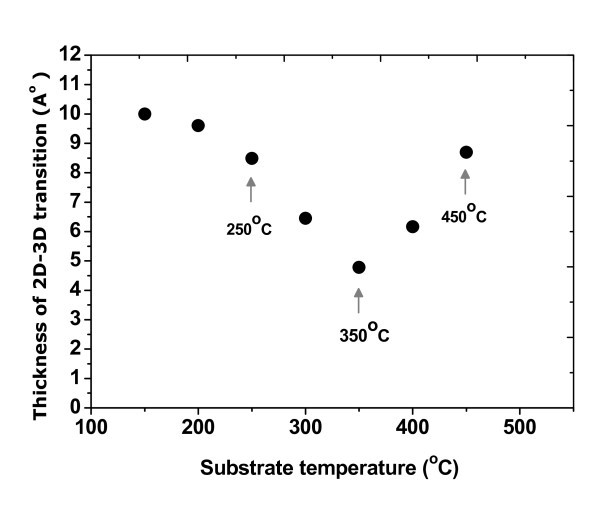
**The dependence of 2D-3D transition thickness during the epitaxy of the Ge_0.96_Sn_0.04 _film on the substrate temperature in the range of 150-450°C**.

The diffraction patterns at the growth process of Ge and Ge_0.96_Sn_0.04 _films on Si(100) were similar. At the first stage of epitaxial growth, the authors observed the diffraction pattern from flat surfaces of the wetting layer and found the pattern to become 3D after the Ge_0.96_Sn_0.04 _layer has grown a few nm larger. By the diffractometry of high energy electrons during the process of epitaxy, the critical thickness can be determined, i.e., the thickness of transition from the 2D growth mode to the 3D growth mode for the heterostructures of Ge_0.96_Sn_0.04_/Si(100), which depends on the growth temperature of substrates. The dependence of 2D-3D transition thickness during the epitaxy of Ge_0.96_Sn_0.04 _film on the substrate temperature in the range of 150-450°C is shown in Figure 1. It can be seen that the temperature dependence has a non-monotonic character with the minimum at 350°C.

**Figure 2:**
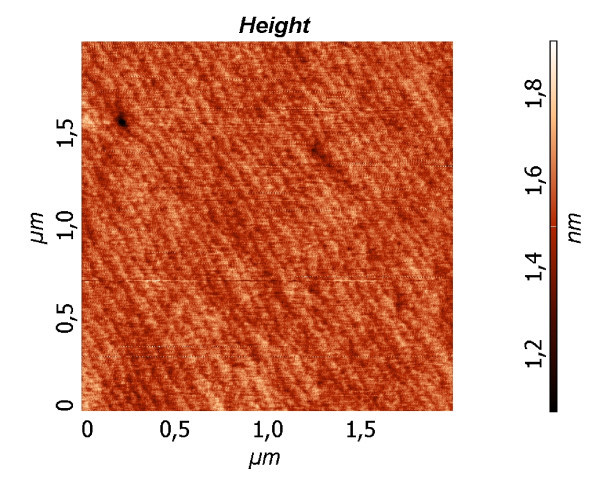
**AFM image from wetting layer Ge_0.96_Sn_0.04 _with 0.33 nm thickness, grown at 350°C**.

Moreover, the oscillations of specular beam of diffraction pattern were not observed during the growth in all the investigated temperature ranges, i.e., 150-450°С. It means that the Ge-Sn films grow by the moving atomic steps on the surface. The result of RHEED was also supported by the AFM and STM measurements. Our MBE system allows one to grow four films with different thicknesses from the wetting layer, and three films with a higher thickness in one process on the same substrate. The micromorphology of all the grown films was studied by AFM and STM. Before 2D-3D transition, one has the flat wetting layer at all substrate temperatures. The wetting layers contain the atomic steps with the edge orientation < 110 >. The typical AFM image of this layer with 0.33 nm thickness is shown in Figure 2. It shows that the root mean square is equal to 0.0955 nm at 350°C.

So far, the nature of nonmonotonic temperature dependence of transition 2D-3D thickness is not clear. It was shown in the article [13], that the mobility of Ge atoms on the Si(111) surface increases by several orders of magnitude with a Sn coverage of about one monolayer. Owing to this fact, the Ge_0.96_Sn_0.04 _films seem to grow by the moving atomic steps at relatively low growth temperatures. As long as Sn atoms in growing surfaces act as surfactants for Ge adatoms, the surface diffusion of Ge atoms on a Si(100) surface will increase. The quantity of Sn atoms at growing surfaces may increase because of the effect of Sn segregation. The characteristics of segregation and temperature dependence of Sn segregation during the growth process of the Ge-Sn film are not found in literature.

**Figure 3:**
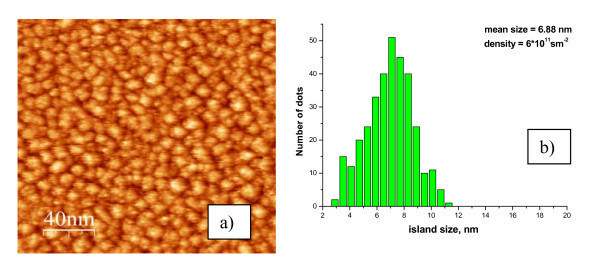
**(a) STM image (200 × 200 nm^2^) from the Ge_0.96_Sn_0.04 _film with 1.08 nm thickness, grown at 250°C**. **(b) **The dependence of quantity ND on the lateral size.

**Figure 4:**
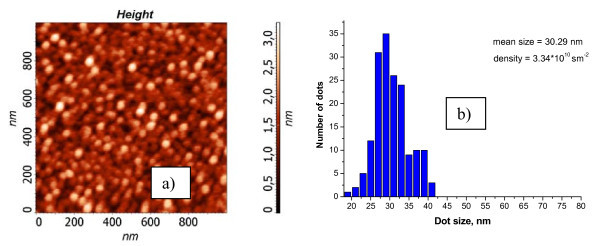
**(a) AFM image (1 × 1 μm^2^) from the Ge_0.96_Sn_0.04 _film with 1.58 nm thickness, grown at 350°C**. **(b) **The dependence of quantity ND on the lateral size.

**Figure 5:**
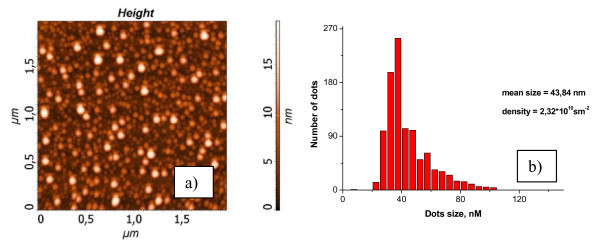
**(a) AFM image (2 × 2 μm^2^) from the Ge_0.96_Sn_0.04 _film with 1.58 nm thickness, grown at 450°C**. **(b) **The dependence of quantity ND on the lateral size.

The 2D RHEED patterns correspond to the flat wetting layer (see Figure 2). The diffraction patterns with 3D spots correspond to AFM images with Ge-Sn islands. The typical STM and AFM pictures are shown in Figures 3, 4, 5. The dependence of ND quantity on the lateral size was calculated for all images. Maximum density of ND (6 × 10^11 ^cm^-2^) with the average lateral size of 7 nm was obtained at 250°C.

**Figure 6:**
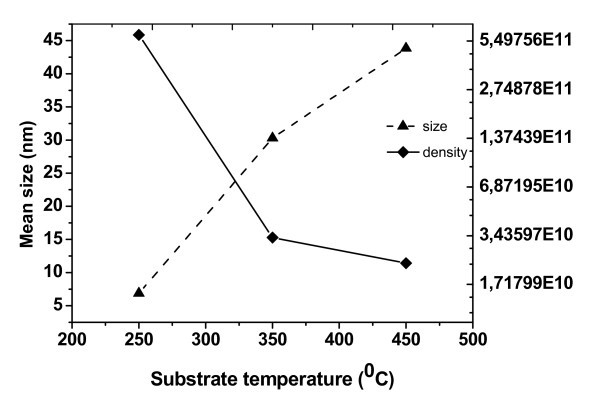
**The dependence of average size of ND and their density on substrate temperatures**.

**Figure 7:**
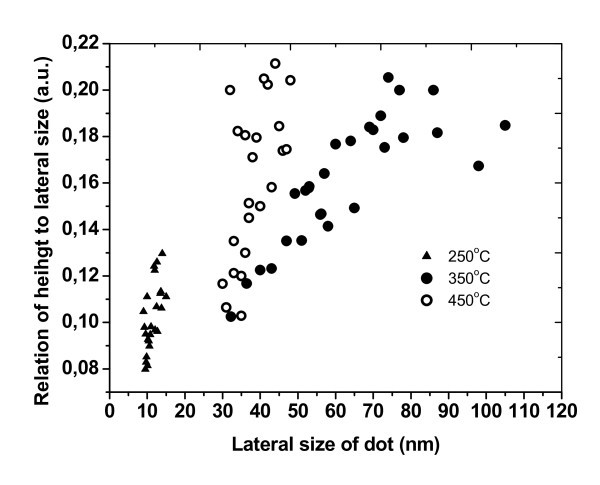
**The dependence of relation of height to lateral size on the lateral size of ND**. Lateral size is equal to square root of the base area.

The dependence of ND of average-size and their density on the growth temperatures is depicted in Figure 6. It can be seen that the average size increases, and the density of ND decreases as the growth temperature increases. The relationship of height to lateral size with the lateral size of ND is shown in Figure 7. This aspect ratio for Ge ND deposited on Si(100) surface is widely reported in the literature. For hut clusters, the aspect ratio is equal to 0.1-0.2 [14,15]. ND grown at the substrate temperature of 250°C have a similar aspect ratio 0.08-0.13 (see Figure 7). It is also found that the Ge_0.96_Sn_0.04 _ND at low temperature of epitaxy have a shape similar to the Ge hut cluster. The nanoislands grown at higher temperatures of the substrate (350-450°C) had a bigger lateral size from 30 to 110 nm and the aspect ratio of ND changed from 0.10 to 0.21. These data characterized the ND with the shape similar to the one of the dome Ge cluster.

## Conclusion

From the data on RHEED, AFM, and STM, it is concluded that molecular beam growth of Ge_1-*x*_Sn*_x _*heterostructures with the small concentrations of Sn in the range of substrate temperatures from 150 to 450°C follows the SK mechanism. By the method of recording diffractometry of high energy electrons during the process of epitaxy, the wetting layer thickness of Ge_0.96_Sn_0.04 _films is found to depend on the temperature of the substrate. The micromorphology of the Ge_0.96_Sn_0.04_/Si(100) heterostructures surface has been investigated in the range of substrate temperatures from 250 to 450°C by AFM and STM *ex situ*. Maximum density of ND (6 × 10^11 ^cm^-2^) with the average lateral size of 7 nm has been obtained at 250°C.

## Abbreviations

AFM: atomic force microscopy; MBE: molecular beam epitaxy; ND: nanodots; RHEED: reflection of high energy electron diffraction; SK: Stranski-Krastanow; STM: scanning tunnel microscopy.

## Competing interests

The authors declare that they have no competing interests.

## Authors' contributions

VM carried out the design of the study and drafted the manuscript, VU carried out the growth experiments in MBE machine, VT performed the statistical analysis of AFM and STM images, AN performed the RHEED analysis and participated in its design, OP performed the STM analysis and participated in its design and coordination, ISY carried out the AFM measurements and participated in its analysis, HC participated in the design of the study and its coordination. All authors read and approved the final manuscript.

## References

[B1] Montragoon P Vukmirović N Ikonić Z Harrison P Electronic structure and optical transitions in Sn and SnGe quantum dots in a Si matrix Microelectron J 2009 40 483

[B2] Montragoon P Vukmirović N Ikonić Z Harrison P Electronic structure and optical properties of Sn and SnGe quantum dots J Appl Phys 2008 103 103712

[B3] Nakamura Y Fujinoki N Ichikawa M Photoluminescence from Si-capped Ge-Sn nanodots on Si substrates formed using an ultrathin SiO_2 _film technique J Appl Phys 2009 106 014309

[B4] Nakamura Y Masada A Cho S-P Tanaka N Ichikawa M Epitaxial growth of ultrahigh density of Ge_1-*x*_Sn*_x _*quantum dots on Si(111) substrates by codeposition of Ge and Sn on ultrathin SiO_2 _films J Appl Phys 2007 102 124302

[B5] Nakamura Y Masada A Ichikawa M Quantum-confinement effect in individual Ge_1-*x*_Sn*_x _*quantum dots on Si(111) substrates covered with ultrathin SiO_2 _films using scanning tunneling spectroscopy Appl Phys Lett 2007 91 013109

[B6] Gurdal O Desjardins P Carlsson JRA Taylor N Radamson HH Sundgren J-E Greene JE Low temperature growth and critical epitaxial thicknesses of fully strained metastable Ge_1-*x*_Sn*_x _*(x < 0.26) alloys on Ge(001) 2 × 1 J Appl Phys 1998 83 162

[B7] Hansen M Anderko K Constitution of Binary Alloys 1958 New York: McGraw-Hill

[B8] Pukite PR Harwit A Iyer SS Molecular beam epitaxy of metastable, diamond structure Sn*_x_*Ge_1-*x *_alloys Appl Phys Lett 1989 54 2142

[B9] Wegscheider W Olajos J Menczigar U Dondl W Abstreiter G Fabrication and properties of epitaxially stabilized Ge/α-Sn heterostructures on Ge(001) J Cryst Growth 1992 123 75

[B10] Stranski IN Krastanow VL Sitzungsber Akad Wiss Wien Math-Naturwiss Kl Abt 2B 1938 146 797

[B11] Brunner K Si/Ge nanostructures Rep Prog Phys 2002 65 27

[B12] Nikiforov AI Ulyanov VV Timofeev VA Pchelyakov OP Wetting layer formation in superlattices with Ge quantum dots on Si(100) Microelectron J 2009 40 782

[B13] Dolbak AE Olshanetsky BZ Effect of adsorbed Sn on Ge diffusivity on Si(111) surface Cent Eur J Phys 2008 6 634

[B14] Kamins TI Carr EC Williams RS Rosner SJ Deposition of three-dimensional Ge islands on Si(001) by chemical vapor deposition at atmospheric and reduced pressures J Appl Phys 1997 81 211

[B15] Baribeau J-M Wu X Rowell NL Lockwood DJ Ge dots and nanostructures grown epitaxially on Si J Phys Condens Matter 2006 18 R139

